# Emerging evidence on noncoding-RNA regulatory machinery in intervertebral disc degeneration: a narrative review

**DOI:** 10.1186/s13075-020-02353-2

**Published:** 2020-11-16

**Authors:** Hao-Yu Guo, Ming-Ke Guo, Zhong-Yuan Wan, Fang Song, Hai-Qiang Wang

**Affiliations:** 1 Department of Orthopaedics, Union Hospital, Tongji Medical College, Huazhong University of Science and Technology, Wuhan, 430022 People’s Republic of China; 2 Department of Orthopaedic Surgery, The Affiliated Hospital of PLA Army Medical University Warrant Officer School, Shijiazhuang, 050000 People’s Republic of China; 3 Department of Orthopedics, The Seventh Medical Center of Chinese PLA General Hospital, Beijing, 100700 People’s Republic of China; 4 Department of Stomatology, PLA Rocket Force Characteristic Medical Center, Beijing, 100088 People’s Republic of China; 5 Institute of Integrative Medicine, Shaanxi University of Chinese Medicine, Xixian Avenue, Xixian District, Shaanxi Province 712046 People’s Republic of China

**Keywords:** Apoptosis, Cell proliferation, Extracellular matrix degeneration, Intervertebral disc degeneration, Inflammation, Noncoding-RNA, Nucleus pulposus cell

## Abstract

Intervertebral disc degeneration (IDD) is the most common cause of low-back pain. Accumulating evidence indicates that the expression profiling of noncoding RNAs (ncRNAs), including microRNAs (miRNAs), circular RNAs (circRNAs), and long noncoding RNAs (lncRNAs), are different between intervertebral disc tissues obtained from healthy individuals and patients with IDD. However, the roles of ncRNAs in IDD are still unclear until now. In this review, we summarize the studies concerning ncRNA interactions and regulatory functions in IDD. Apoptosis, aberrant proliferation, extracellular matrix degradation, and inflammatory abnormality are tetrad fundamental pathologic phenotypes in IDD. We demonstrated that ncRNAs are playing vital roles in apoptosis, proliferation, ECM degeneration, and inflammation process of IDD. The ncRNAs participate in underlying mechanisms of IDD in different ways. MiRNAs downregulate target genes’ expression by directly binding to the 3′-untranslated region of mRNAs. CircRNAs and lncRNAs act as sponges or competing endogenous RNAs by competitively binding to miRNAs and regulating the expression of mRNAs. The lncRNAs, circRNAs, miRNAs, and mRNAs widely crosstalk and form complex regulatory networks in the degenerative processes. The current review presents novel insights into the pathogenesis of IDD and potentially sheds light on the therapeutics in the future.

## Background

Intervertebral disc degeneration (IDD) is the most common cause of low-back pain, which affects over 70% of people at some points of their whole lifetime [1–3]. However, due to the poor understandings of the pathogenesis of the disorder, few treatment regimens have been put forward, and none of the current clinical interventions for IDD has been confirmed as efficient and radical treatment modalities [3–5]. Therefore, an in-depth investigation of the regulatory machinery of IDD is urgently needed in the present.

**Fig. 1:**
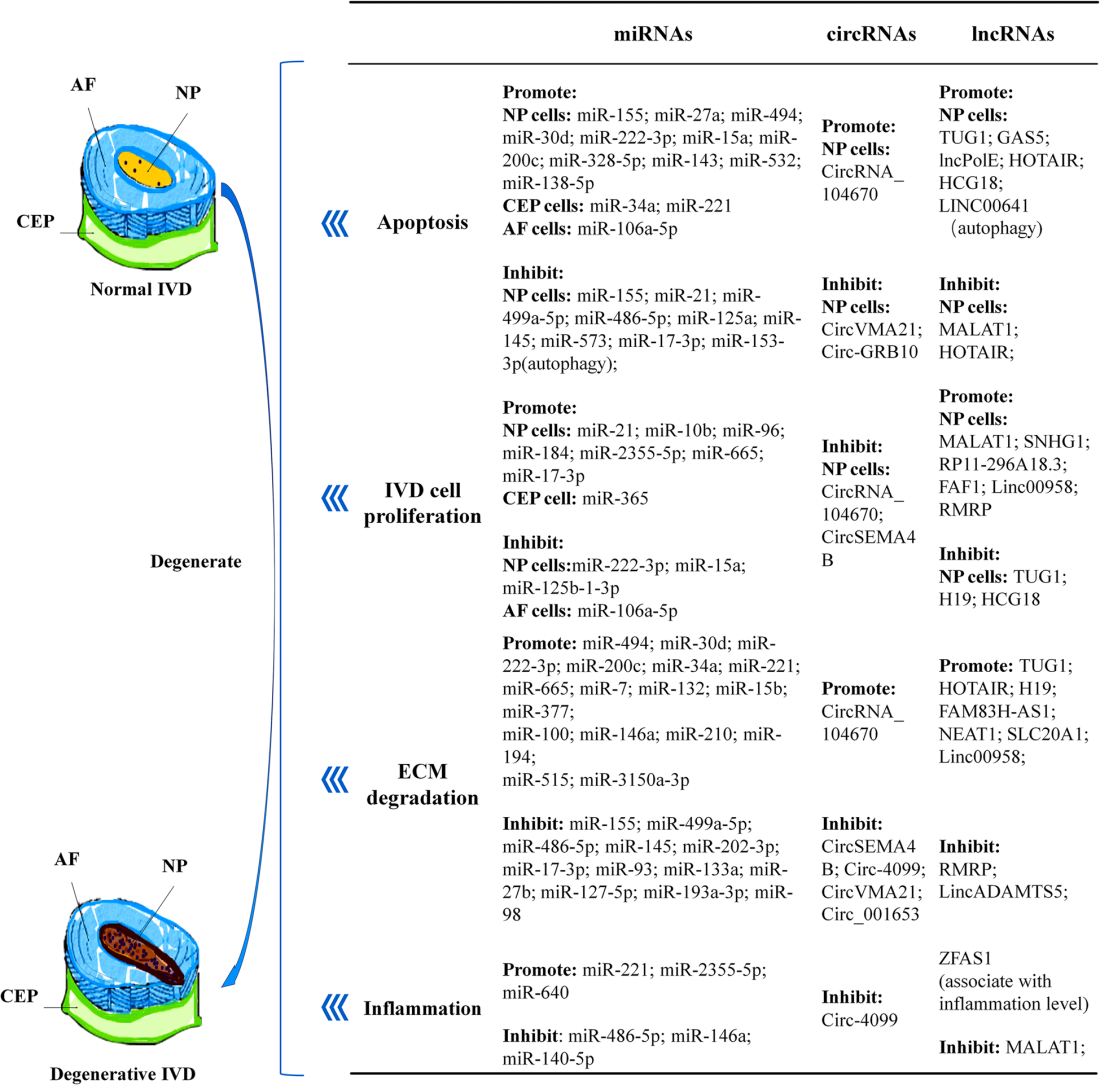
Noncoding RNAs involved in multiple pathological processes of IDD development (apoptosis, ECM degradation, cell proliferation, and inflammation)

Intervertebral disc (IVD) can be divided into three morphologically distinct regions, i.e., the sandwiched central nucleus pulposus (NP), peripheral annulus fibrosus (AF), and cranial or caudal cartilaginous endplate (CEP) (Fig. 1). During the process of IDD, the apoptosis of IVD cells is abnormally increased with the cells aberrantly clustering, dysregulation of extracellular matrix (ECM) proteins (abnormally synthesized and/or degraded), and excessive expression of inflammatory factors which accelerate the formation of inflammatory microenvironment/niche and eventually violate the adjacent IVD cells [6–10]. These pathophysiological processes result in a vicious circle of progressive aggravation of degeneration.

Emerging evidence reveals that genetic and environmental factors are both influencing factors of IDD, whereas genetic factors seem to be the outweighed one. Notably, a multitude of genetic factors, implicating in the underlying regulatory mechanisms, are dysregulated in IDD, especially the noncoding RNAs (ncRNAs) [6, 11, 12]. NcRNAs consist of a large family of RNAs without coding function and outcome as cellular effectors, i.e., proteins. So far, the identified ncRNAs in *homo* *sapiens* include miRNAs, circRNAs, lncRNAs, and emerging small RNAs. The expression profiling of ncRNAs of IDD samples is significantly different from those from healthy ones, reflected by differentially expressed levels and types of ncRNAs unraveled by microarray and/or sequencing analyses. It is suggested that ncRNAs are playing vital roles in apoptosis, proliferation, ECM degeneration, and inflammation process of IDD [12–17]. Owing to that, we established the coding-noncoding SuperSeries Datasets as GSE67567 in human IDD, including lncRNAs, mRNAs, and circRNAs, and miRNAs datasets as GSE19943, GSE63492, GSE56081, and GSE67566, as well as studies from other investigators (Table 1). Given the scarcity of studies summarizing the research progress of ncRNAs in IDD, we designed and conducted a review across the published papers [13, 17, 18, 20–25]. In the current work, the state-of-art research advance and therapeutic potentials concerning the regulatory roles of miRNAs/circRNAs/lncRNAs in degenerated discs of human or animal models were summarized and discussed (Fig. 1).

**Table 1:** mRNA and ncRNA expression profiles of IDD deposited in gene expression omnibus

Data accession number	Types of RNA profiling	Platform	BioProject	Samples	Control set	Publication year	Contributors
**GSE19943**	miRNAs	GPL9946 Exiqon human miRCURY LNA™ microRNA Array V11.0	PRJNA120173	GSM498350 GSM498351 GSM498352 GSM498353 GSM498354 GSM498355)	3 control (scoliosis) vs. 3 degenerative nucleus pulposus (NP) cell samples, extracted from NP tissue without cultures	2011	Wang et al. [18]
**GSE45856**	miRNAs	GPL11434 miRCURY LNA microRNA Array, 6th generation - hsa, mmu & rno	PRJNA196506	GSM1116694 GSM1116695 GSM1116696 GSM1116697 GSM1116698 GSM1116699	3 control (traumatic normal) vs. 3 degenerative IVD tissues using TRIspin method	2013	Zhao et al. [19]
**GSE56081**	mRNAs LncRNAs	GPL15314 Arraystar Human LncRNA microarray V2.0 (Agilent_033010 Probe Name version)	PRJNA242356	GSM134764 GSM134765 GSM134766 GSM134767 GSM134768 GSM134769 GSM1347770 GSM134771 GSM134772 GSM134773	5 control (cadaveric normal) vs. 5 degenerative NP tissues using TRIspin method	2014	Wan et al. [13]
**GSE63492**	miRNAs	GPL19449 ExiqonmiRCURY LNA microRNA Array miRBase v18.0	PRJNA268036	GSM1551024 GSM1551025 GSM1551026 GSM1551027 GSM1551028 GSM1551029 GSM1551030 GSM1551031 GSM1551032 GSM1551033	5 control (cadaveric normal) vs. 5 degenerative NP tissues using TRIspin method	2016	Lan et al. [20]
**GSE67566**	circRNAs	GPL19978 Agilent-069978 Arraystar Human CircRNA microarray V1	PRJNA280274	GSM1649704 GSM1649705 GSM1649706 GSM1649707 GSM1649708 GSM1649709 GSM1649710 GSM1649711 GSM1649712 GSM1649713	5 control (cadaveric normal) vs. 5 degenerative NP tissues using TRIspin method	2016	Lan et al. [20]
**GSE67567**	Noncoding RNA SuperSeries	GPL15314 Arraystar Human LncRNA microarray V2.0 (Agilent_033010 Probe Name version) GPL19449 GPL19978	PRJNA280271	In combination	5 control (cadaveric normal) vs. 5 degenerative NP tissues	2016	Lan et al. [20]
**GSE153761**	lncRNAs, mRNAs and circRNAs	GPL22120 Agilent-078298 human ceRNA array V1.0 4X180K	PRJNA643990	GSM4653870	3 control (traumatic normal) vs. 3 degenerative cartilage endplate of cervical disc	2020	Yuan et al.
				GSM4653871			
				GSM4653872			
				GSM4653873			
				GSM4653874			
				GSM4653875			

## The regulatory mechanism of miRNAs in IDD

### The expression profile and molecular mechanisms of miRNAs in IDD

Accumulating evidence indicates that the miRNA expression profile in IDD cases is significantly different from those in the controls. In 2011, we presented the first line of evidence on miRNA expression profiling in IDD, using scoliosis NP tissues as control. Twenty-nine differentially expressed miRNAs were identified, with 6 upregulated and 23 downregulated [18]. Thereafter, the emerging molecules as miRNAs catch the attention of global researchers, manifesting as an increasing number of published studies. Subsequently, Zhao et al. compared the expression profile of miRNAs between IDD and spinal cord injury patients in 2014. Twenty-six miRNAs were downregulated in the IDD group, while 25 upregulated [19]. Further investigation revealed these dysregulated miRNAs controlled several signaling pathways, which are pivotal in the pathogenesis of IDD, such as Wnt [19, 26, 27], phosphoinositide 3-kinase/Akt (PI3K/Akt) [19, 28], and mitogen-activated protein kinase (MAPK) [19, 29], etc. Consistently, Hu et al. demonstrated that among the 253 miRNAs detected both in IDD and scoliosis samples, three were downregulated and six were upregulated in degenerative samples. The downstream targets were predicted to be genes or proteins associated with degeneration, such as drosophila mothers against decapentaplegic protein family member 4 (SMAD4), which play important roles in cell-cycle-related pathways [30].

Complementary base sequence endows miRNAs the ability to bind the 3′untranslated region (3′UTR) of particular mRNA. The binding of miRNAs and mRNAs results in a decreased expression of the target proteins [31, 32], while most of them are hub proteins, which play a crucial role in essential pathways associated with degeneration. Thus, miRNAs indirectly control the pathological processes in disc degeneration. The IDD-related miRNAs are presented in Table 2.

**Table 2:** Experimentally verified miRNAs associated with IDD

MiRNA	Expression	Target(s)	Functions	Publication year	References
MiR-155	↓	FADD, caspase-3 ERK1/2, TCF7L2, MMP-16	↓NP cell apoptosis ↓ECM degradation	2011 2016–2018	[18] [33–35]
MiR-21	↓* ↑	PTEN PTEN, PDCD4 PTEN	↓NP cell apoptosis ↑NP cell proliferation ↑ECM degradation	2018 2014,2016 2018	[36] [21, 37] [38]
MiR-27a	↑	PI3K	↑NP cell apoptosis	2013	[39]
MiR-499a-5p	↓	SOX4	↓NP cell apoptosis ↓ECM degradation	2019 2019	[40] [40]
MiR-494	↑	SOX9, JunD SOX9	↑NP cell apoptosis ↑ECM degradation	2015,2017 2017	[41, 42] [41]
MiR-30d	↑	SOX9	↑NP cell apoptosis ↑ECM degradation	2018 2018	[43] [43]
MiR-222-3p	↑	CDKN1B	↑NP cell apoptosis ↓NP cell proliferation ↑ECM degradation	2019 2019 2019	[44] [44] [44]
MiR-15a	↑	MAP3K9	↑NP cell apoptosis ↓NP cell proliferation	2017 2017	[45] [45]
MiR-486-5p	↓	FOXO1	↓NP cell apoptosis ↓ECM degradation ↓inflammation	2019 2019 2019	[46] [46] [46]
MiR-200c	↑	XIAP	↑NP cell apoptosis ↑ECM degradation	2018 2018	[23] [23]
MiR-328-5p	↑	ERBB2	↑NP cell apoptosis	2018	[47]
MiR-34a	↑	GDF5 Bcl-2	↑ECM degradation ↑CEP cell apoptosis	2016 2015	[48] [7]
MiR-143	↑	Bcl-2	↑NP cell apoptosis	2017	[49]
MiR-532	↑	Bcl-9	↑NP cell apoptosis	2018	[50]
MiR-125a	↓	TP53INP1	↓NP cell apoptosis	2016	[51]
MiR-221	↑	ERα FOXO3, TRPS1 BMP-Smad pathway	↑CEP cell apoptosis ↑ECM degradation ↑inflammation ↓chondrogenesis ↓AF cell osteogenic differentiation	2018 2018 2018 2018 2016	[52] [52] [52] [53] [54]
MiR-138-5p	↑	SIRT1	↑NP cell apoptosis	2016	[55]
MiR-145	↓	ADAM17	↓NP cell apoptosis ↓ECM degradation	2019 2019	[56] [56]
MiR-573	↓	Bax	↓NP cell apoptosis	2019	[57]
MiR-153-3p	↓	ATG5	↓NP cell autophagy	2019	[58]
MiR-106a-5p	↑	ATG7	↑AF cell apoptosis ↓AF cell proliferation	2019 2019	[8] [8]
MiR-10b	↑	HOXD10	↑NP cell proliferation	2013	[59]
MiR-96	↑	ARID2	↑NP cell proliferation	2017	[60]
MiR-184	↑	GAS1	↑NP cell proliferation	2017	[61]
MiR-2355-5p	↑	ERFFI1	↑NP cell proliferation ↑inflammation	2019 2019	[62] [62]
MiR-365	↓	HDAC4	↑CEP cell proliferation	2019	[63]
MiR-125b-1-3p	↑	TSHZ3	↓NP cell proliferation	2018	[64]
MiR-665	↑	GDF5	↑NP cell proliferation ↑ECM degradation	2018 2018	[65] [65]
MiR-7	↑	GDF5	↑ECM degradation	2016	[66]
MiR-132	↑	GDF5	↑ECM degradation	2017	[67]
MiR-15b	↑	SMAD3	↑ECM degradation	2017	[68]
MiR-20a	↑	ANKH	↑CEP chondrocyte calcification	2016	[69]
MiR-377	↑	ADAMTS5	↑ECM degradation	2013	[70]
MiR-202-3p	↓	MMP1	↓ECM degradation	2019	[71]
MiR-17-3p	↓	MMP2	↓ECM degradation ↓NP cell apoptosis ↑NP cell proliferation	2018 2018 2018	[72] [72] [72]
MiR-93	↓	MMP3	↓ECM degradation	2015	[73]
MiR-133a	↓	MMP9	↓ECM degradation	2016	[74]
MiR-27b	↓	MMP13	↓ECM degradation	2016	[75]
MiR-127-5p	↓	MMP13	↓ECM degradation	2017	[76]
MiR-193a-3p	↓	MMP14	↓ECM degradation	2016	[77]
MiR-98	↓	IL-6	↓ECM degradation	2016	[78]
MiR-100	↑	FGFR3	↑ECM degradation	2015	[79]
MiR-146a	Not clear**	TRAF6	↑ECM degradation ↓inflammation	2015 2015,2017	[22] [22, 80]
MiR-210	↑	ATG7	↑ECM degradation	2017	[81]
MiR-194	↑	CHSY1/2/3	↑ECM degradation	2017	[82]
MiR-515	↑	CHSY1/2/3	↑ECM degradation	2017	[82]
MiR-3150a-3p	↑	ACAN	↑ECM degradation	2018	[83]
MiR-640	↑	LRP1,β-catenin, EP300	↑inflammation	2019	[84]
MiR-140-5p	↓	TLR4	↓inflammation	2018	[85]

The expression, targets, and functions of miRNAs related to IDD were displayed in Table 2. “↓” represents downregulation, while “↑” represents upregulation

*Decrease in apoptotic NP cells.**It is reported that miR-146a is significantly downregulated in the PBMCs of IDD patients, but its expression in NP cells is unclear [80]

In summary, 49 miRNAs were reported with a relationship to IDD, among the total number of 38,589 miRNAs of *Homo sapiens*, according to miRBase Release 22.1 (http://www.mirbase.org/). Whereas studies have been focused on intra-cellular miRNAs, cell-free miRNAs emerge as potential novel biomarkers for a variety of human diseases. Recently, exRNA Atlas has been proposed across human biofluids, which is also essential in the regulation of IDD [86].

### The roles of miRNAs in IVD cell apoptosis

Accumulating evidence shows that several miRNAs function as inducers or inhibitors in the apoptosis of IVD cells via specific target genes or pathways [87]. For instance, downregulated miR-155 was suggested triggering the Fas-mediated apoptosis by disinhibiting *FADD* and *CASP-3* in NP cells [18]. Similarly, the expression of miR-21 [36], miR-499a-5p [40], miR-486-5p [46], miR-125a [51], miR-145 [56], and miR-573 [57] are decreased in IDD, which act as apoptosis inhibitors via binding to the 3′UTRs of mRNAs of *PTEN*, *SOX4*, *FOXO1*, *TP53INP1*, *ADAM17*, and *Bax*, respectively. In contrast to these findings, miRNAs such as miR-27a [39], miR-494 [41, 42], miR-30d [43], miR-222-3p [44], miR-15a [45], miR-143 [49], miR-532 [50], miR-138-5p [55] in NP cells, miR-106a-5p [8] in AF cells, and miR-34a [7] and miR-221 [52] in CEP cells, display potential pro-apoptotic effects in IDD, via inhibiting the expression of downstream hub proteins in several pathways. Apart from the aforementioned mechanisms, miRNAs, i.e., miR-153-3p, participating in the autophagy, also contributes to the disc degeneration eventually [58].

In summary, there are eight miRNAs acting as inhibitors of apoptosis in IDD, whereas eleven miRNAs act as promoters of apoptosis.

### The roles of miRNAs in IVD cell proliferation

Cell number in healthy human IVDs is limited and sparsely distributed. However, the cells were reported to proliferate into clusters in IDD [88]. In this complex pathophysiological process, multiple miRNAs acting as vital indirect regulators in IVD cell proliferation can be employed as biomarkers. For example, in NP cells, the aberrant overexpression of miR-21 increases the proliferation level of degenerated NP cells by downregulating *PDCD4* and *PTEN*. Thus, the disinhibition effect increased the phosphorylation level of c-Jun and AKT proteins, which could induce cell proliferation. Liu et al. found that miR-21 knockdown reversed cell proliferation, while *Ly294002*, an *AKT* inhibitor, reversing the effect induced by miR-21. These results indicate that miR-21 is a potential biomarker and therapeutic target of IDD [21, 37].

Besides, overexpression of miR-10b [59], miR-96 [60], miR-184 [61], miR-2355-5p [62], and miR-665 [65] could also promote the proliferation of degenerated NP cells via targeting *PTEN/PDCD4*, *HOXD10*, *ARID2*, *GAS1*, *ERFFI1*, and *GDF5*, while upregulation of miR-222-3p [44], miR-15a [45], and miR-125b-1-3p [64] had an opposite effect by inhibiting the expression of *CDKN1B*, *MAP3K9*, and *TSHZ3*. These downstream genes regulate NP cell proliferation by controlling crucial pathways, such as RhoC-Akt pathway [59], PTEN/AKT pathway [21, 37], ARID2/AKT signaling [60], and activating/deactivating molecular molecules like AKT [21, 37, 61]. Among them, miR-222-3p promotes the proliferation of IVD cells and accelerates the apoptosis and ECM degradation via the same pathway [44]. In accordance with this, miR-15a [45], miR-106a-5p [8] and miR-17-3p [72] have a similar effect, which limits their application as therapeutic targets.

In addition to NP cells, miR-106a-5p [8] in AF cells and miR-365 [63] in CEP cells are also associated with cell proliferation, by inhibiting the proliferation level via *ATG7* and increasing proliferation via *HDAC4*, respectively.

Collectively, there were 12 miRNAs involved in IVD cell proliferation, with eight miRNAs promoting proliferation and four miRNAs inhibiting proliferation.

### The roles of miRNAs in ECM degradation and inflammation

Generally, IVD cells play an essential role in secreting ECM components like collagens and proteoglycans to maintain IVD’s structural stability and resist mechanical loads [89, 90]. However, in IDD, the unbalance between synthesis and degradation of ECM makes the IVD unrenewable and degenerative, especially in NP tissues [91]. MiRNAs modulate the degradation of ECM by regulating the expression of essential enzymes such as matrix metalloproteinases (MMPs) or cytokines such as interleukins.

It is reported that inhibition of miR-665 [65], miR-7 [66], miR-132 [67], and miR-34a [48] effectively attenuate ECM degradation in degenerative NP tissues by directly upregulating the expression of growth differentiation factor-5 (GDF5), which can inhibit the expression of ECM catabolic factors, such as *MMP* and *ADAMTS4*, and upregulating the production of anabolic proteins, such as type II collagen and aggrecan.

A series of miRNA, i.e., miR-202-3p [71], miR-17-3p [72], miR-93 [73], miR-133a [74], miR-27b [75], miR-127-5p [76], miR-193a-3p [77], and miR-155 [33] are significantly downregulated in degenerative NP tissues, with their expression levels reversely correlated with the grade of IDD, which induce type II collagen synthesis via directly suppressing the expression levels of *MMP1*, *MMP2*, *MMP3*, *MMP9*, *MMP13*, *MMP14*, and *MMP16*, respectively, whereas overexpression of miRNAs mentioned above can stop and reverse the degradative process, indicating that they are potential biomarkers and therapeutic targets of IDD.

In addition, two different protective mechanisms of miR-155 have been clarified in ECM degradation. Ye et al. have shown that the knockdown of miR-155 results in decreased expressions of collagen II and glycosaminoglycan by increasing the expression of *ERK1/2* [34]. Sun et al. have reported that an essential transcription factor, *TCF7L2*, which acts as an activator in the process of chondrocyte matrix degradation through *p65*/*NF-κB* signaling, was repressed by miR-155 [35].

Wang et al. discovered that miR-21 is upregulated in IDD tissues and positively correlated with the degradation grade, which indicates miR-21 cannot only inhibit NP cell apoptosis and promote proliferation as mentioned above, but also promote ECM degradation through repressing the *PTEN*/*AKT*/*mTOR* signaling pathway [38]. SRY-related high-mobility group box (*SOX*)*-4* and *SOX9* are respectively targeting molecules of miR-499a-5p [40], miR-494 [41], and miR30d [43], by repressing the apoptosis of NP cells and ECM degradation.

As well, a number of miRNAs can affect the process of ECM degradation, including miR-222-3p [44], miR-486-5p [46], miR-221 [52, 53], miR-145 [56], and miR-98 [78] in NP tissues, miR-221 [54] in AF tissues, and miR-20a [69] in CEP tissues. The expression, targets, and functions of these miRNAs are listed in Table 2.

Apart from apoptosis, proliferation, and ECM degradation, inflammation responses and inflammatory cytokines are also regarded as crucial factors in the pathogenesis of IDD [92]. miRNAs associated with the production of inflammation cytokines, such as miR-486-5p [46], miR-221 [52], miR-2355-5p [62], miR-146a [22, 80], miR-640 [84], and miR-140-5p [85] are also listed in Table 2 and can also be used as therapeutic targets of IDD. In general, there are six reported miRNAs pertaining to inflammation during IDD in various subparts of the IVDs via a multitude of targeting genes, affecting a variety of inflammatory cytokines. Three deregulated miRNAs (miR-140-5p targeting *TLR4* [85], miR-486-5p targeting *FOXO1* [46] and miR-146a targeting *TRAF6* [22, 80]; all studying in NP cells) are associated with decreased levels of inflammation, whereas three miRNAs (miR-221 targeting *ERα* in CEP cells [52], miR-640 targeting *LRP1*, *β-catenin* and *EP300* in NP and AF cells [84], and miR-2355-5p targeting *ERFFI1* in NP cells [62]) are linked with increased levels of inflammation during IDD.

## The regulatory mechanism of circRNAs in IDD

### The profile and mechanism of circRNAs in IDD

CircRNAs are a group of single-stranded RNAs with loop structures, which act as competing endogenous RNAs (ceRNAs) and restore the functions of specific genes by sponging miRNAs [17, 93]. A specific miRNA could be sponged by various distinct circRNAs, forming a circRNA-miRNA-mRNA interaction network [20, 47, 72]. Thus, circRNAs seems like a critical regulator in gene expression.

We presented the first line of evidence of circRNAs expression profiling in human IDD In 2016. We found 636 differentially expressed circRNAs in human lumbar IVDs, with 354 upregulated and 282 downregulated [20]. Zou and colleagues indicated that many genes regulated by circRNAs are playing crucial roles in the pathogenesis of IDD, via over 15 signaling pathways, such as Wnt and integrin signaling pathways. Pairs of host genes and circRNA can be divided into four categories according to their profile: circRNA and its host genes downregulated, circRNA and its host genes upregulated, circRNA downregulated and its host genes upregulated, and circRNA upregulated and its host genes downregulated [93].

Several experiments were conducted to investigate the differences between the profile of circRNAs in degenerative IVDs and that in normal IVDs. Wang et al. have provided another line of evidence that 72 circRNAs were upregulated by more than two-fold in degenerative NP tissues [94]. Following this, another team identified there were 7294 circRNAs aberrantly expressed (3724 upregulated, 3570 downregulated, fold change > 2) in degenerative NP cells [17]. Recently, Li et al. reviewed the results from related publications from 2016 to 2019 and confirmed that the profile in IDD patients is different from that in the control group, with the number of upregulated circRNAs ranging from 51 to 3724, and the number of downregulated circRNAs ranging from 21 to 3570 [15].

As the dynamic development of miRBase reflecting novel findings in miRNAs, multiple circRNA databases have been proposed with changing numbers and updated findings as well. So far, there are hundreds of human circRNAs reported (148 in chondrocytes and 104 in osteocytes) [95].

### The roles of circRNAs in IDD

Specific circRNA can indirectly regulate apoptosis, proliferation, and ECM degradation by modulating the level of functional miRNA, contributing to the disc degeneration. Specifically, CircVMA21 [23], Circ-GRB10 [47], and CircRNA_104670 [72] are involved in apoptosis regulation. CircRNA_104670 [72] and CircSEMA4B [96] are related to NP cell proliferation. CircVMA21 [23], Circ-4099 [94], CircSEMA4B [96], and CircRNA_104670 [72] are associated with ECM degradation.

As shown in Table 3, miR-200c accelerates the apoptosis of NP cell and ECM degradation via inhibiting *XIAP*, whereas CircVMA21 alleviates the negative effect of sponging miR-200c. However, in degenerative IVD tissues, the expression of CircVMA21 is repressed, resulting in aberrant higher level of miR-200c and IDD [23]. Circ-GRB10 is downregulated in degenerative NP tissues. Transient overexpression of GRB10 could attenuate the apoptosis of NP cells by sequestering miR-328-5p and leading to the activation of genes associated with proliferation via the ErbB pathway [47].

**Table 3:** Experimentally verified circRNAs associated with IDD

CircRNA	Expression	Target miRNA	Functions	Publication year	References
CircVMA21	↓	miR-200c	↓NP cell apoptosis ↓ECM degradation	2018 2018	[23] [23]
Circ-GRB10	↓	miR-328-5p	↓NP cell apoptosis	2018	[47]
CircSEMA4B	↓	miR-431	↓NP cell proliferation ↓ECM degradation	2018 2018	[96] [96]
CircRNA_104670	↑	miR-17-3p	↑NP cell apoptosis ↓NP cell proliferation ↑ECM degradation	2018 2018 2018	[72] [72] [72]
Circ-4099	↑	miR-616-5p	↓ECM degradation ↓Inflammation	2018 2018	[94] [94]

The expression, targets, and functions of circRNAs related to IDD were displayed in Table 3. “↓” represents downregulation, while “↑” represents upregulation

Besides, overexpression of circSEMA4B could inhibit NP cells from proliferation and ECM degradation induced by *IL-1β* via indirectly rescuing SFRP1 or GSK-3β in Wnt signaling from miR-431 [96]. Song et al. found that upregulated CircRNA_104670 accelerates apoptosis and inhibits cell proliferation and collagen II synthesis in NP cells via circRNA_104670/miR-17-3p/MMP2 network [72]. In contrast, upregulated Circ-4099 acts as a protective factor by disinhibiting the expression of collagen II and aggrecan and downregulating the synthesis of the pro-inflammatory factors such as *IL-1β*, *TNF-α*, and *PGE2* by sponging miR-616-5p. The expression data, targeted miRNAs, and functions of circRNA in IDD are listed in Table 3.

## The regulatory mechanism of lncRNAs in IDD

### The profile and mechanism of lncRNAs in IDD

LncRNAs are a group of ncRNAs with more than 200 nucleotides. LncRNAs take the role of ceRNAs (as circRNAs) or small interfering RNA (siRNAs) and participate in the lncRNA/circRNA/miRNA/mRNA network as transcriptional regulators [97]. They regulate gene expression or control the signaling pathways by competitively sponging and inactivating specific miRNAs [12, 13]. Some lncRNAs even regulate the activity or stability of proteins by directly interacting with them [98, 99]. Investigations indicate that lncRNAs exert their regulatory function in various ways (i.e., reducing the methylation level of the promoter region may accelerate the expression of specific lncRNAs in IVD cells [100]). Therefore, the aberrant expression of lncRNAs will cause the degeneration of IVD cells and result in the development of IDD.

Ample evidence indicates that the profile of lncRNAs in degenerative IVDs is totally different from those in normal IVDs. In 2014, we reported the first expression profiling of lncRNAs in human IDD by using the same human lumbar IVD samples as circRNAs. One hundred sixteen lncRNAs (with 67 upregulated and 49 downregulated) and 260 mRNAs were differentially expressed in degenerative samples with an absolute fold change greater than ten [13]. Among the deregulated lncRNAs in IDD, HOTAIR (NR_003716) is the top downregulated lncRNAs (fold change, 148.53; *P* < 0.001) [13]. Later, Zhao et al. reported that 1530 of 1854 differential expressed lncRNAs might have 6386 potential target genes, whereas Han et al. reported 632 lncRNAs are differentially expressed in IDD tissues among 40,716 detected lncRNAs [101, 102]. Li and colleagues reviewed the articles related to expression profiles of lncRNAs and summarized the number of differentially expressed lncRNAs. The number of upregulated lncRNAs is ranging from 67 to 2234, while the downregulated ones ranging from 49 to 938 [97]. These results indicate that lncRNAs could modulate the destiny of NP cells in IDD and be transformed into screening biomarkers or therapeutic targets.

### The roles of lncRNAs in IDD

The roles of lncRNAs in IDD can be divided into four main categories according to their functions (apoptosis, cell proliferation, ECM degradation, inflammation) as well. A specific lncRNA can have two or more functions simultaneously.

Chen et al. found that overexpression of *TUG1* in degenerative NP samples accelerates cell apoptosis, via upregulating the levels of *Bax*&caspase-3 (the latter are pro-apoptotic factors) in Wnt1/β-catenin pathway and downregulating the levels of *Bcl-2*, an anti-apoptotic factor. In addition, the increased level of *TUG1* also deteriorates the degradation of ECM by breaking the expression balance in the ECM-degrading and anti-ECM-degrading genes [24]. Both GAS5 and lncPolE are overexpressed in degenerative IVD samples, displaying similar roles in apoptosis. While GAS5 increases the apoptosis by binding to miR-155, lncPolE negatively regulates *PolE* [100, 103].

Emerging evidence suggests that autophagy is an essential process in IDD and has a close relationship with apoptosis. Zhang and colleagues reported that overexpression of HOTAIR accelerates NP cell apoptosis via stimulating cell autophagy [104]. On the contrary, Shao et al. indicated that downregulated HOTAIR expression inhibits cell apoptosis via the Notch signaling pathway by sponging miR-34a-5p. In other words, the overexpression of HOTAIR reduces NP cell apoptosis [105]. On account of the incompatible viewpoints, further investigations are needed to clarify the real effects of HOTAIR in apoptosis. In addition to HOTAIR, LINC00641 accelerates cell autophagy by sponging miR-153-3p, which can inactivate autophagy-related gene 5 (*ATG5*) [58].

Aberrant cell proliferation is another core pathogenesis in IDD. SNHG1 promotes NP cell proliferation via sponging miR-326, and downregulated miR-326 disinhibits NP cell proliferation by inactivating *PCNA* and cyclin D1 expression. Similarly, RP11-296A18.3/miR-138/HIF1A, RMRP/miR-206/PCNA, H19/miR-22/LEF1/Wnt/β-catenin signaling, and HCG18/miR-146a-5p/TRAF6/NF-κB axis can also increase or decrease the level of proliferation, respectively [10, 25, 106–108]. Targeting extracellular signal-regulated kinase (Erk) and miR-146a-5p/TRAF6/NF-κB axis, respectively, lncRNA FAF1 and HCG18 modulate the ratio of synthesis-phase cells among all the cells in NP tissue [109].

H19, Linc00958, and SLC20A1 have been reported to upregulate ECM degradation via sponging miRNAs [107, 109, 110]. It is noteworthy that H19 plays a role as a competitor to LEF1 for binding miR-22, regulating Wnt/β-catenin pathway [107]. Linc00958 and NEAT1 exert their function by increasing the expression of MMPs via upregulating SMAD and inhibiting the synthesis of aggrecan and collagen-II in the ERK/MAPK pathway, respectively [109, 111]. Wei et al. demonstrated that decreased FAM83H-AS1 in IDD results in ECM degeneration, by targeting Notch1 and Hes1 [112]. While Linc-ADAMTS5, interacting with splicing factor proline/glutamine-rich (SFPQ), which induces the down expression of ADAMTS5, alleviates the ECM deterioration process [113].

Inflammatory cytokines and inflammatory cytokine-related lncRNAs are also involved in IDD. Several members of the interleukin family, such as IL-1 and IL-6, were widely noted as pro-inflammatory factors, giving rise to the degeneration of ECM and apoptosis of IVD cells. In vitro studies showed that overexpression of MALAT1 attenuates IL-1 and IL-6 induced inflammation by sponging miR-503, displaying a protective effect on IVD cell [114]. Besides, ZFAS1 is linked with inflammatory cytokine levels in IDD. Since the positive correlation between the intensity of inflammatory and severity of degeneration, ZFAS1 is regarded as a sensitive predictor of IDD [115].

LncRNAs related to the modulation and prediction of IDD are listed in Table 4.

**Table 4:** Experimentally verified lncRNAs associated with IDD

LncRNA	Expression	Target(s)	Functions	Publication year	References
TUG1	↑	Wnt1/β-catenin, Bax& caspase-3	↑NP cell apoptosis ↓NP cell proliferation ↑ECM degradation	2017 2017 2017	[24] [24] [24]
GAS5	↑	miR-155	↑NP cell apoptosis	2019	[103]
LncPolE	↑	PolE	↑NP cell apoptosis	2019	[100]
HOTAIR	↑ ↓	AMPK/mTOR/ULK1 miR-34a-5p	↑NP cell apoptosis ↑ECM degradation ↓NP cell apoptosis	2020 2020 2019	[104] [104] [105]
MALAT1	↓	miR-503	↓NP cell apoptosis ↑NP cell proliferation ↓Inflammation	2017 2017 2017	[114] [114] [114]
LINC00641	↑	miR-153-3p	↑NP cell autophagy	2019	[58]
SNHG1	↑	miR-326	↑NP cell proliferation	2018	[106]
RP11-296A18.3	↑	miR-138	↑NP cell proliferation ↑ECM synthesis	2017 2017	[10] [10]
H19	↑	miR-22	↓NP cell proliferation ↑ECM degradation	2018 2018	[107] [107]
FAF1	↑	Erk	↑NP cell proliferation	2018	[116]
FAM83H-AS1	↑	Notch1	↑NP cell growth ↑ECM degradation	2019 2019	[112] [112]
HCG18	↑	miR-146a-5p	↑NP cell apoptosis ↓NP cell proliferation	2017 2017	[108] [108]
Linc00958	↑	miR-203	↑NP cell proliferation ↑ECM degradation	2019 2019	[109] [109]
RMRP	↑	miR-206	↑NP cell proliferation ↓ECM degradation	2018 2018	[25] [25]
NEAT1	↑	ERK1/2, MAPK	↑ECM degradation	2018	[111]
SLC20A1	↑	miR-31-5p	↑ECM degradation	2019	[110]
Linc-ADAMTS5	↑	SFPQ	↓ECM degradation	2017	[113]
ZFAS1	↑		Associate with inflammation level	2019	[115]

The expression, targets, and functions of lncRNAs related to IDD were displayed in Table 4. “↓” represents downregulation, while “↑” represents upregulation

## Conclusion

In recent years, a large number of investigations have depicted a bright future for ncRNAs, which play roles as delicate regulators in the pathogenesis of IDD. The lncRNA/circRNA/miRNA/mRNA networks and the widespread crosstalks between the RNAs provide us another way to recognize and understand the pathogenesis of IDD [19]. A number of aberrantly expressed RNAs have been regarded as early diagnostic biomarkers or useful therapeutic targets. Moreover, novel materials and technologies, such as injectable hydrogel or nanoparticle which is loadable for small RNAs [117], genetic technologies and stem cell-based therapies [118, 119], are developing rapidly, making it possible to interfere the RNA expression inside IVD cells.

The rapid development of high throughput biotechnological tools greatly facilitates the studies for ncRNAs in IDD. The most common biotechnological approaches are microarray analysis for specific ncRNAs and/or sequencing technologies. Following successful RNA isolation and quality control, ncRNA expression in IDD can be detected via developed microarray chips with known covered ncRNA numbers and types according to corresponding ncRNA database versions. Alternatively, ncRNA expression in IDD can be studied using next-generation sequencing platforms following reverse transcription to cDNA. Thereafter, sequencing data can be mapped to human genomic version (the updated version as GRCh38) and annotated into various subtypes of ncRNAs, with the pros of uncovering novel ncRNAs and cons as introducing errors/mutations during reverse transcription. A combined exploration of both biotechnologies might overcome the cons and improve the studies of ncRNAs in IDD. Novel sequencing technologies are needed for direct sequencing of RNAs and omitting the reverse transcription step. In addition, there are triple common tools/techniques for ncRNA studies following screening. First, RT-PCR tool aims for the detection of expression levels of ncRNAs. Second, bioinformatics and online software tools apply for ncRNA function, target, and interaction predictions. Third, in vitro modulation (upregulation and repression) designates for target and function validations.

However, we are still facing with lots of challenges. Lack of knowledge about the overall view of the ncRNA networks makes it challenging to identify the key nodes to interfere with. The roles of tRNAs and emerging small RNAs, i.e., small nucleolar RNAs (snoRNAs) and PIWI-interacting RNAs (piRNAs), which may be equally important in IDD, remain unclear and deserve thorough studies. Stem cells, such as mesenchymal stem cells (MSCs), have already been used in IVD degeneration therapies for assisting tissue regeneration and exosome secretion, which contains miRNAs to improve microenvironment. However, the inflammatory milieu of IVDs is tough for MSCs to survive in degenerative IVD tissues [120, 121]. Thus, improvement in tissue engineering techniques is urgently needed in seed cell implanting [122]. Future studies should keep focusing on the molecular mechanisms of crosstalk among ncRNAs, especially novel snoRNAs, piRNAs, and tRNAs, and seek feasible ways in seed cell implantation, nanoparticles containing RNA molecules or engineered tissues to interfere the hub nodes in the regulatory network. With the issues solved, research advances in the regulatory machinery of ncRNAs will provide the medical community with a brighter future for IDD therapies.

## Data Availability

All data generated or analyzed during this study are included in this published article [and its supplementary information files].

## Abbreviations

3′UTR: 3′-Untranslated region
ADAMTS: A disintegrin and metalloprotease with thrombospondin motifs
AF: Annulus fibrosus
CDKN1B: Cyclin-dependent kinase inhibitor 1B
CEP: Cartilaginous endplate
CeRNAs: Competing endogenous RNAs
CircRNA: Circular RNA
Col I: Type I collagen
Col II: Type II collagen
ECM: Extracellular matrix
ERα: Estradiol receptor
FADD: Fas-associated death domain-containing protein
FGFR3: Fibroblast growth factor receptor-3
HOXD10: Homeobox D10
IDD: Intervertebral disc degeneration
IL-1β: Interleukin-1β
IVD: Intervertebral disc
lncRNA: Long noncoding RNA
MAPK: Mitogen-activated protein kinase
miRNAs: MicroRNAs
MMPs: Matrix metalloproteinases
NcRNAs: Noncoding RNAs
NP: Nucleus pulposus
PDCD4: Programmed cell death 4
PI3K: Phosphoinositide 3-kinase
PTEN: Phosphate and tension homology deleted on chromosome ten
SAA1: Serum amyloid A1
SMAD4: Mothers against decapentaplegic protein family member 4
SOX: SRY-related high-mobility group box
TGF-β: Transforming growth factor-β
TNF-α: Tumor necrosis factor-α
TRAIL: Tumor necrosis factor-related apoptosis-inducing ligand
